# A Possible Role for Integrin Signaling in Diffuse Axonal Injury

**DOI:** 10.1371/journal.pone.0022899

**Published:** 2011-07-22

**Authors:** Matthew A. Hemphill, Borna E. Dabiri, Sylvain Gabriele, Lucas Kerscher, Christian Franck, Josue A. Goss, Patrick W. Alford, Kevin Kit Parker

**Affiliations:** Disease Biophysics Group, School of Engineering and Applied Sciences, Wyss Institute of Biologically Inspired Engineering, Harvard University, Cambridge, Massachusetts, United States of America; University of Pennsylvania, United States of America

## Abstract

Over the past decade, investigators have attempted to establish the pathophysiological mechanisms by which non-penetrating injuries damage the brain. Several studies have implicated either membrane poration or ion channel dysfunction pursuant to neuronal cell death as the primary mechanism of injury. We hypothesized that traumatic stimulation of integrins may be an important etiological contributor to mild Traumatic Brain Injury. In order to study the effects of forces at the cellular level, we utilized two hierarchical, *in vitro* systems to mimic traumatic injury to rat cortical neurons: a high velocity stretcher and a magnetic tweezer system. In one system, we controlled focal adhesion formation in neurons cultured on a stretchable substrate loaded with an abrupt, one dimensional strain. With the second system, we used magnetic tweezers to directly simulate the abrupt injury forces endured by a focal adhesion on the neurite. Both systems revealed variations in the rate and nature of neuronal injury as a function of focal adhesion density and direct integrin stimulation without membrane poration. Pharmacological inhibition of calpains did not mitigate the injury yet the inhibition of Rho-kinase immediately after injury reduced axonal injury. These data suggest that integrin-mediated activation of Rho may be a contributor to the diffuse axonal injury reported in mild Traumatic Brain Injury.

## Introduction

Blast-induced mild Traumatic Brain Injury (mTBI) is the most frequent wound of the conflicts in Afghanistan and Iraq [1]. Approximately 60% of total combat casualties are associated with blast events generated by improvised explosive devices, and recent studies suggest that nearly 16% of US combatants have been diagnosed with mTBI [2]. Although how blast energy is transmitted to the brain is not well understood, *in vivo* studies and clinical reports have shown that exposure to blast can cause mTBI [2], [3], [4]. Interestingly, the neuronal injury observed in these studies resembles diffuse axonal injury (DAI), a common pathology observed following mTBI *in vivo* [5]. Diffusion tensor imaging studies have identified structural alteration in white matter tracts in military personnel who previously suffered blast-induced mTBI [6], [7], and experimental models have linked these structural alterations to DAI [8]. However, the cellular mechanisms which initiate this pathophysiological response are not well understood.

*In vitro* models of TBI may not fully recapitulate the complexity of the brain, but they provide unique insight into its cellular pathology. Previous models of mTBI have proposed that a disruption in ion homeostasis initiates a sequence of secondary events ultimately leading to neuronal death, however, membrane poration can only account for a portion of injured neurons [9], [10], and excitotoxicity due to changes in ion channel homeostasis [11] cannot account for observations of axonal retraction.

We hypothesized that mechanical perturbation of integrins in the neuronal membrane may represent an injury pathway that would account for DAI in mTBI. Integrins are transmembrane proteins that couple the cytoskeleton in the intracellular space to the matrix network in the extracellular space, providing mechanical continuity across the membrane [12]. Mechanical forces propagating through these coupled networks can activate signal transduction pathways, alter ion channel currents, and initiate pathological cascades [13], [14]. In the brain, integrin signaling is implicated in development and memory potentiation [15], [16], [17], [18], [19], [20], however, there are no reports on the role of integrin signaling in mTBI.

To test our hypothesis, we built a high velocity tissue stretcher to deliver an abrupt mechanical perturbation to cultured neonatal rat cortical neurons. These experiments demonstrated that neuronal injury is a function of focal adhesion size and density. Using magnetic tweezers and coated paramagnetic beads bound to neurons, we measured the difference in the failure strengths of focal adhesions in the soma versus neurites, and found the latter to have significantly weaker attachments to the substrate. Using the magnetic tweezers, we applied an abrupt force to these neurons and found that with fibronectin (FN)-coated beads neurite focal swelling, including abrupt mechanical failure in neurites, occurred 100s of microns away from the soma, suggesting that injury forces may propagate through the neuronal cytoskeleton. Conversely, poly-L-lysine (PLL)-coated beads attached to neurites induced only a local injury. Membrane poration was only observed at extreme strains in a subset of experiments, whereas at lower strains, integrin-induced focal swelling was observed without membrane poration. The injury was not mitigated with the use of a calpain inhibitor, suggesting a calpain-independent injury mechanism. Treatment with a Rho-kinase inhibiter decreased neuronal injury, suggesting a role for downstream integrin-mediated cascade events in neuronal injury.

## Results

### High Speed Stretch Induces Strain-Dependent Neuronal Injury

**Figure 1:**
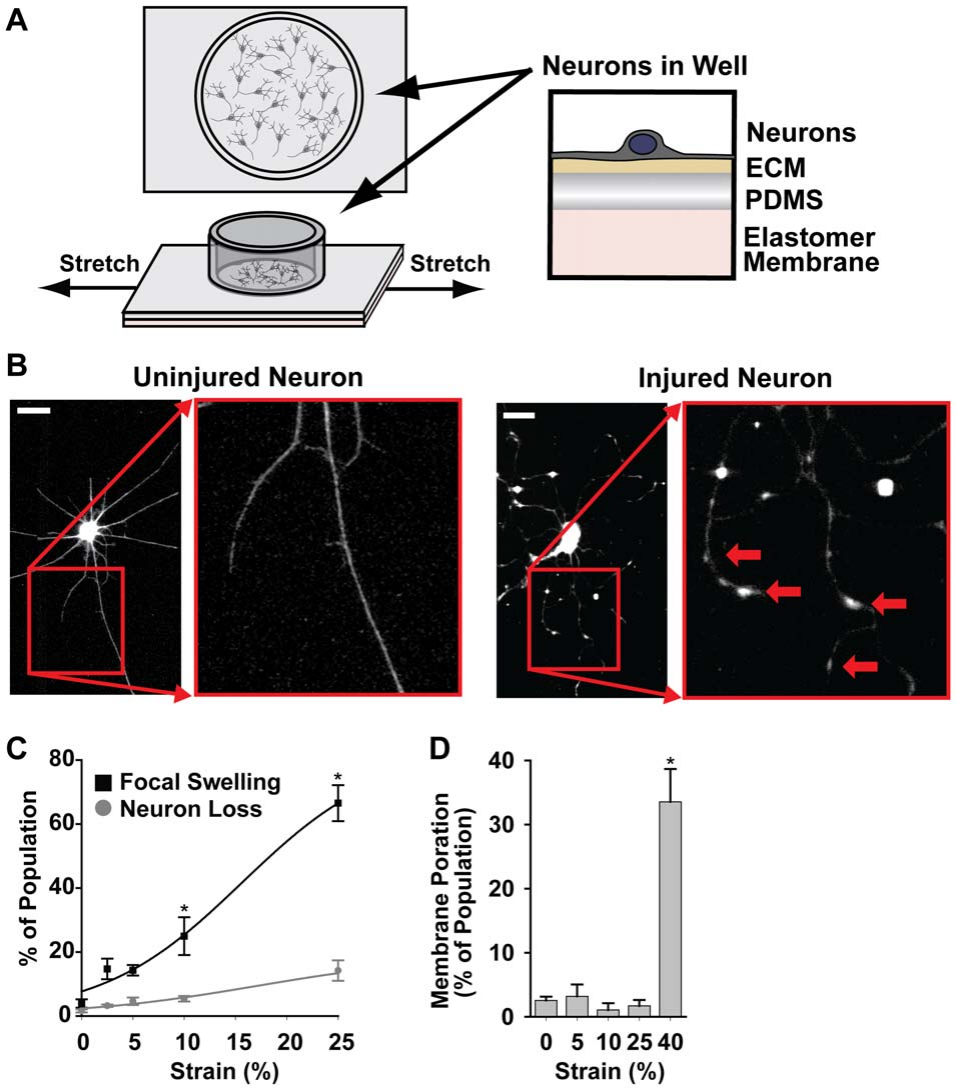
High speed stretch model of neuronal cultures indicates a strain dependent injury response identified by focal swelling of the neurites without porating the membrane. (**A**) Neurons were cultured on elastomer membranes that were quickly stretched, transferring injurious forces to neurons. (**B**) Beta-3-Tubulin immunofluorescence imaging showed that prior to stretch, neurons exhibited a highly branched, smooth neurite morphology. After stretch, many neurons developed widespread focal swellings along their neurites (red arrows) (Scale Bar  = 20 µm). (**C**) Quantification of neuronal injury showed an initial significant response between 0% and 10% strains (*n*≥4). Neuron loss due to stretch also increased with strain magnitude. (**D**) The percentage of neurons exhibiting signs of membrane poration, as indicated by the uptake of a membrane impermeable dye, following stretch showed an initial significant increase between 25% and 40% strain (*n*≥3). All bars SEM for all panels, * p<0.05.

The spatio-temporal profile of the mechanical perturbation, such as a blast wave, in the brain is likely variable and, given the timescale of blast wave propagation, quite rapid. In order to mimic this sudden mechanical stimulus, we designed and built a high speed stretcher (HSS) system to deliver an abrupt strain to a population of neurons cultured on a flexible silicon elastomer substrate coated with PLL (Fig. 1A), similar to previous *in vitro* stretch models [21]. We seeded primary neonatal rat cortical neurons on stretchable membranes five days before experiments to allow dendritic and axonal extension. During experiments, the substrates underwent an abrupt, uniaxial stretch (at 1% per ms) to generate a strain field of defined magnitude (Fig. S1 and Video S1). Neuronal injury was defined as the appearance of focal swellings along neurites, neurite retraction, or abrupt mechanical failure of the neurite (Fig. 1B), similar to injury morphologies reported in previous *in vitro* fluid shear models of injury [9] and similar to swelling observed in DAI *in vivo* [22]. We found that neuronal response to stretch was heterogeneous and dependent upon strain magnitude (Fig. 1C), similar to what has been reported *in vivo* [10]. Few neurons were lost, defined as abrupt failure of all attachment to the substrate, due to the stretch at strain magnitudes less than 10% and a small increase in loss was observed at 25% strain. At 10 minutes following stretch, a significant increase in focal swelling was observed for strain magnitudes greater than 5%. For all subsequent studies, we focused on strain magnitudes of 0**–**10%, as this range captured the threshold of inducing neuronal injury. Also, in this strain range only a small percentage of neurons exhibited signs of mechanoporation, as indicated by the uptake of membrane impermeable dye from the extracellular solution (Fig. 1D), or apoptosis, as indicated by TUNEL staining (Fig. S2). Thus, we identified a strain dependent injury response in our neuronal populations that is not explained by membrane poration.

### Stretch Injury is Focal Adhesion Complex (FAC) Density-Dependent

**Figure 2:**
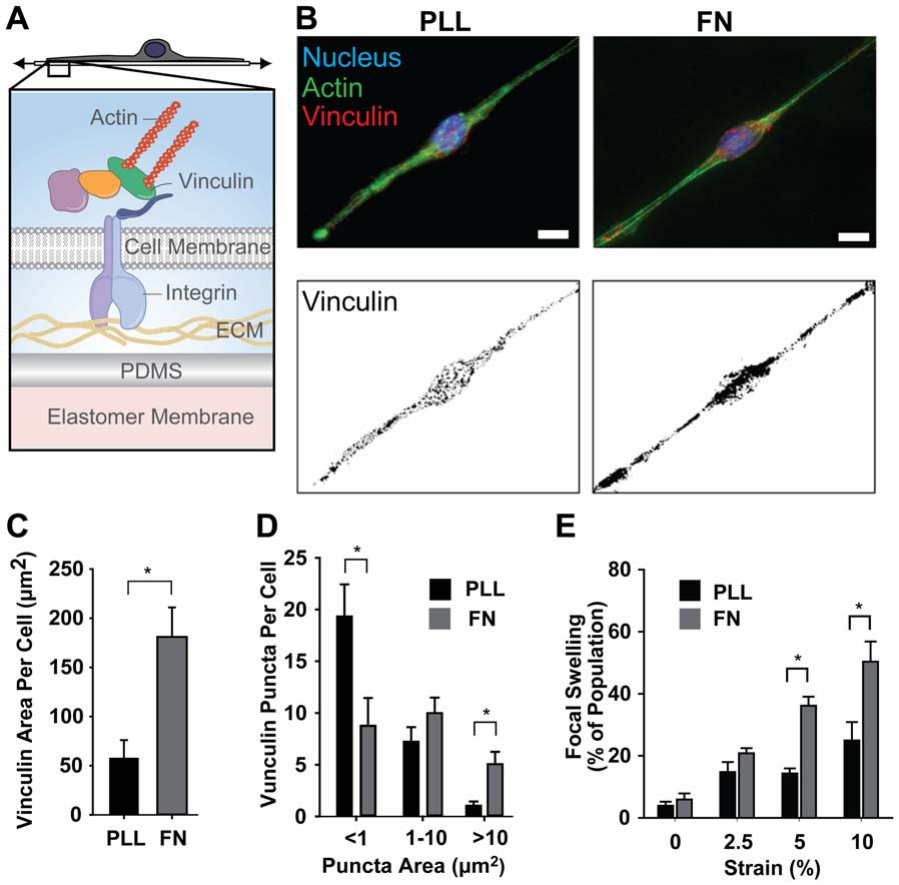
Substrate coating influences neuronal FAC formation and injury progression. (**A**) Neurons are mechanically coupled to the substrate via FACs that couple the intracellular cytoskeleton to the ECM. (**B**) Immunofluorescent imaging of vinculin puncta indicated the presence of FACs. Scale bars correspond to 8 and 10 µm, for PLL and FN respectively. Quantification of (**C**) total vinculin puntca area (*n* = 8) (**D**) indicated that a FN coated substrate induced FAC formation over a larger area and with greater average cluster size compared to a PLL coated substrate (*n* = 5). (**E**) The percentage of neurons that exhibited widespread focal swelling following stretch injury was greater on a FN coated substrate compared to a PLL coated substrate at 10 minutes (*n*≥4 for PLL and *n*≥8 for FN). All bars SEM for all panels, * p<0.05.

The cytoskeleton of the neuron is anchored to the substrate through FACs [23] providing a link for force propagation in the cell (Fig. 2A). We reasoned that we could control FAC density by culturing neurons on microcontact printed lines (10 µm wide) of PLL or FN to guide neurite extension. On PLL surfaces, extracellular matrix (ECM) deposition from media serum provides specific attachment sites for neuronal FACs (Fig. 2B, S3). By using vinculin as a marker for FACs, we measured total FAC area in each cell and found that neurons cultured on FN-coated substrates formed significantly more FACs per cell (181±30 µm^2^) than PLL-coated substrates (58±18 µm^2^) (Fig. 2C). Furthermore, analyzing individual regions (puncta) of FACs revealed that FACs were also smaller and less dense per unit area on PLL-coated substrates as compared to those in neurons on FN-coated substrates (Fig. 2C–D).

We asked how neuronal focal adhesion density affected the neuronal injury. We coated the culture wells of the stretchable substrates with either FN or PLL prior to seeding them with neurons to regulate the density and number of FACs. After five days in culture, we subjected the neuronal networks to an abrupt strain with the HSS system. We observed an increase in the proportion of neurons exhibiting focal swellings on FN-coated substrates when compared to neurons cultured on PLL at both 5% and 10% strains (Fig. 2E). Since PLL-coated substrates induce the formation of smaller and less dense FACs when compared to neurons cultured on FN, the difference in injury rates as a function of FAC size and density suggests a role for an integrin-mediated injury mechanism. In this case, abrupt stretch of the cell substrate uniformly injures the more robust focal adhesion architectures of the FN-seeded neurons because they are more rigidly adhered at networked points throughout the neuron's soma and neurites.

### Neurites Are More Susceptible to Injury

**Figure 3:**
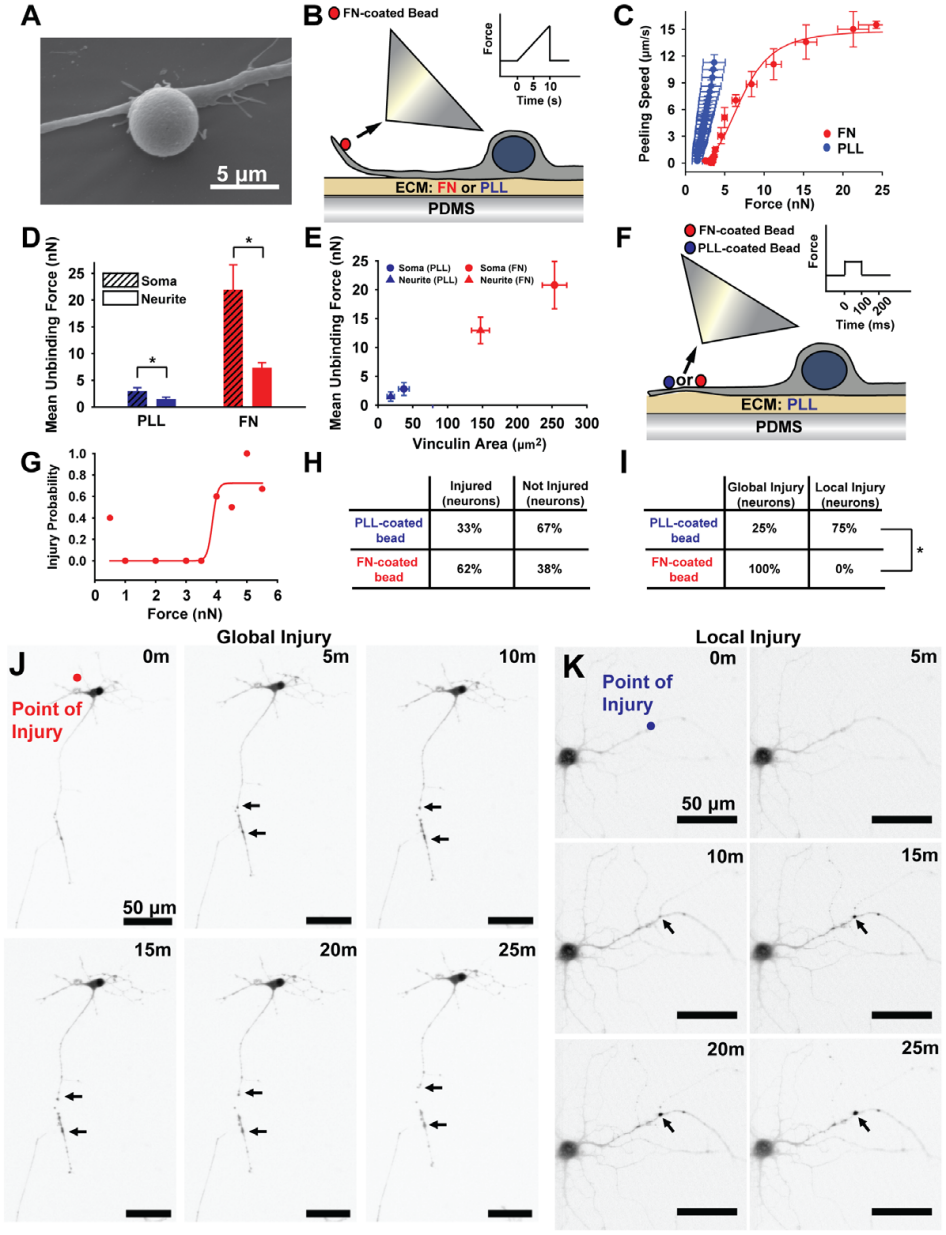
Role of integrins in adhesion strengthening and injury. (**A**) Paramagnetic beads, as shown by SEM, were bound to neurons. (**B**) The failure strength of neuron/substrate adhesions was measured using either FN-coated (red) or PLL-coated (blue) substrates. The beads were pulled with an ascending ramp in force as indicated by the inset. (**C**) The speed at which neurons detached from the substrate (Peeling Speed) during the ascending pull was plotted as a function of the applied force for PLL-coated (blue) and FN-coated substrates (red) (*n*≥4). (**D**) The maximum force required for complete detachment (Mean Unbinding Force) for soma (dashed) and neurite (plain) was plotted for PLL-coated substrates (blue) and FN-coated substrates (red) (*n*≥4). (**E**) Mean unbinding forces for the soma (circles) and neurites (triangles) of cells on PLL or FN coated substrates was plotted as a function of mean vinculin area (*n*≥4). (**F**) Magnetic Tweezers were used to deliver a 100 ms pulse (inset) to neurons with either FN (red) or PLL (blue) coated beads. (**G**) FN-coated beads were used to establish an injury dose response curve. (**H**) FN-coated beads were able to injure cells more often than PLL-coated beads and the extent of injury (I) depended upon bead coating. (**J**) FN-coated beads always caused global cellular injury (focal swellings indicated by black arrows extended throughout the cell), while (**K**) PLL-coated beads tended to injure locally to the bead-pull site (*n* = 13 for FN-coated beads and *n* = 12 for PLL-coated beads). Inverted fluorescence images from neurons loaded with Fluo-4 calcium dye. All bars SEM for all panels, * p<0.05.

Given the focal nature of axonal swelling in DAI [22], it is reasonable to assume that there is heterogeneous vulnerability to injury within the various structures of a neuron, such as the dendrites, axons, and soma. Examination of FAC density in immunostained neurons led us to hypothesize that the larger, more numerous FACs of the soma would endow it with a higher threshold for mechanical failure than those in neurites. We used magnetic tweezers to apply nanoNewton (nN) forces to 4.5 µm FN-coated paramagnetic beads bound to specific segments of individual neurons (Fig. 3A). By increasing the applied force with time (Fig. 3B), we peeled neurons from the PLL and FN coated substrates. After correcting for displacement of the paramagnetic bead position relative to the magnetic tweezer tip, we found a linear behavior in the speed with which neurons were peeled from PLL-coated substrates whereas neuronal peeling on FN-coated substrates was represented by a sigmoidal curve (Fig. 3C). These differences can be directly related to the FAC density (see Fig. 2B) and thus suggest adhesion strengthening on FN-coated substrates. We sought to determine the relative vulnerabilities of the soma versus the neurite to strain injury and compared the failure strengths of FACs in these different regions. We reasoned that a relative difference in FAC failure strength between the soma and its neurites would serve as an indicator of vulnerability to mechanical injury. We used the magnetic tweezers to measure the maximum force required to break the FACs that bound the soma and neurites to the substrate. The force required to detach the soma was found to be higher than that required to detach the neurite for both coatings, and significantly larger for FN-coated substrates (Fig. 3D). The contribution of vinculin-containing FACs in the adhesion strengthening of the soma versus the neurite is illustrated by the linear relationship between mean unbinding force and focal adhesion size (Fig. 3E). The differences in adhesion strength suggest that axonal and dendritic extensions have a vulnerability to integrin-mediated mechanical injury in axons.

### Injury Extent Depends on Integrin Binding

Integrins provide mechanical continuity between the ECM and the cytoskeleton, thus mediating the possible propagation of mechanical forces bidirectionally across the membrane. The cytoskeleton is an integrated polymer network that propagates mechanical forces throughout a cell. We asked whether a brief, traumatic pull to simulate injury forces via integrins (FN-coated paramagnetic beads), versus a nonspecific (PLL-coated paramagnetic beads) administration of the force to the cell, would result in different injury modalities (Fig. 3F). We reasoned that this experiment would reveal an injury threshold, similar to the force thresholds previously reported for integrin-mediated neurite formation [24]. Using magnetic tweezers, we administered abrupt (100 msec), 0.5**–**5.5 nN forces to FN-coated paramagnetic beads attached to the surfaces of cultured neurons and established an injury force dose response curve. These data revealed a focal adhesion injury threshold of 4nN (Fig. 3G). Consistent with an integrin-mediated injury mechanism, 62% (*n* = 13) of neurons were injured with FN-coated beads, while 33% (*n* = 12) of neurons were injured with PLL-coated beads (Fig. 3H), in agreement with the results reported in Fig. 2E with the HSS. In neither case was membrane poration observed (Fig. S4). The ability of PLL-coated beads bound to the apical surface of the axon to injure despite their inability to specifically bind integrins was likely due to the fact that neurons attach to the substrate through integrins on the basal surface and local stretching of the cell membrane may activate these integrin complexes and induce injury, albeit at a lower rate. Furthermore, abrupt pull of bound FN-coated beads consistently induced formation of focal swellings on neurites extending from the opposite side of the soma, generating a global injury (100% of injured neurons, Fig 3I and Video S2), where focal swellings appeared up to 150 µm away from the bead pull site (Fig 3J and Fig. S5). Similar perturbations of PLL-coated beads tended to injure near the point of attachment, generating a local injury (Fig. 3K and Video S3). We also tested a 1 sec bead pull and noted similar injury morphologies (Fig. S6). It should be noted that neither the magnetic field alone, attached beads alone, nor Acetylated-LDL-coated beads were able to induce injury (Figs. S6 **–** S8). That integrin-bound beads were able to injure neurons globally, while PLL-coated beads tended to injure cells only locally, suggests that despite the local nature of the insult, integrin-mediated forces result in injury at a distance, leading to a global, cellular response propagated through the CSK.

### Injury is ROCK-Dependent

**Figure 4:**
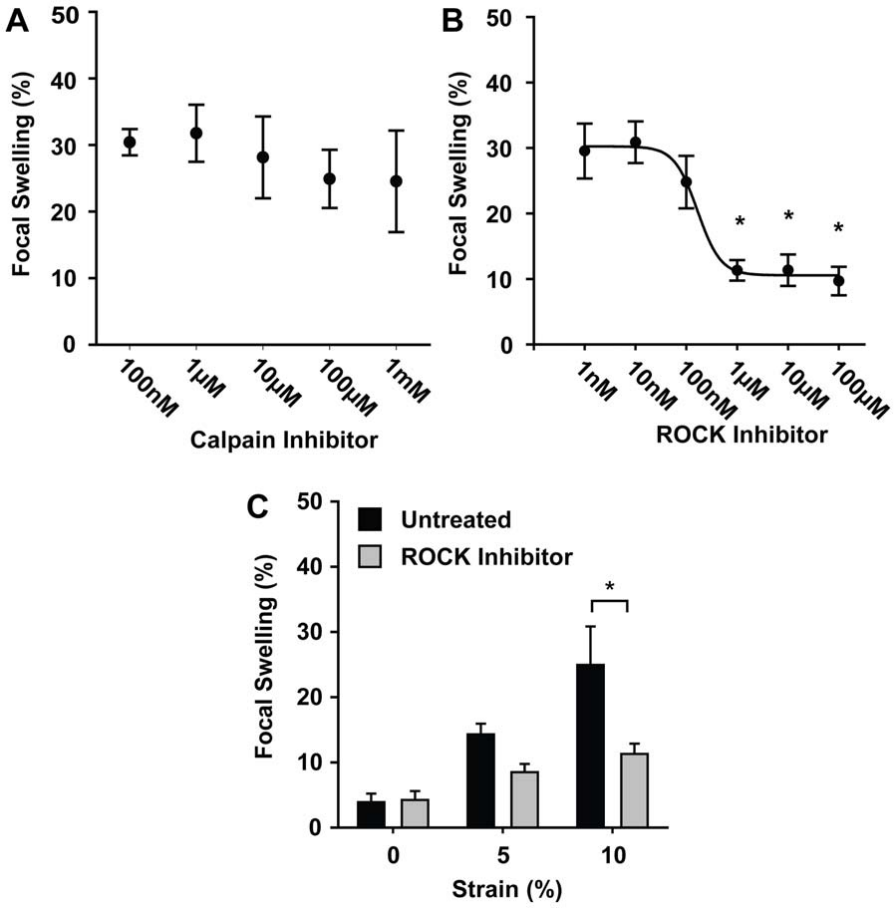
Pharmacological inhibition of secondary injury pathways may reduce neuronal injury. (**A**) Immediate administration of a Calpain inhibitor MDL 28170 following 10% stretch of neurons seeded on PLL substrates was unable to reduce the percentage of injured neurons 10 minutes later (*n*≥4). (**B**) However, immediate application of a ROCK inhibitor, HA-1077, was able to reduce neuronal injury in a dose dependent manner (*n*≥5). (**C**) Decreases in injury were observed at both 5% and 10% strain magnitude (*n*≥5). All bars SEM for all panels, * p<0.05.

Integrin signaling may activate secondary signaling cascades which cause neuronal injury. Previous reports suggest that cysteine proteases, such as calpains, actively degrade the cytoskeleton and that their inhibition can reduce neuronal injury [10], [25]. Others, however, have suggested the involvement of additional or multiple pathways leading to different forms of neuronal injury [26], [27]. We asked if a calpain inhibitor would reduce the instance of focal swelling in our model. Using the HSS system with neuronal cultures seeded on PLL substrates, we observed that the application of MDL-28170 to inhibit calpain activation either before (Fig. S9), or immediately following, abrupt stretch yielded no significant change in neurite focal swelling, suggesting that calpain activation cannot explain neuronal injury in our model (Fig. 4A). Previous work has shown that integrin mediated RhoA activation may cause cytoskeleton reorganization, stiffening, and contraction in other cell types [28], [29]. Since increased RhoA activity has been noted in previous *in vivo* TBI models [30], and more recently inhibition of ROCK, a downstream effector of RhoA, has been shown to be an important therapeutic target in various neurodegenerative disease [31], we asked whether integrin-activated Rho-ROCK signaling may contribute to neuronal injury in our model. Immediate application of HA-1077, a ROCK inhibitor, following stretch with the HSS system resulted in a dose-dependent decrease in the percentage of neurons exhibiting focal swellings (Fig 4B). This apparent neuroprotective effect of HA-1077 was observed at both 5% and 10% strain magnitudes (Fig 4C). These studies suggest that an integrin-mediated signaling cascade may be converging on a ROCK-mediated pathway, identifying a series of potential targets for future *in vivo* therapeutic studies.

## Discussion

Here we have shown that an acute mechanical perturbation of neuronal integrins is sufficient to induce neuronal focal swelling, reminiscent of DAI *in vivo*. Previous studies have attributed this injury to a loss of ionic homeostasis caused by either a disruption of the cell membrane [9], [10], [32] or changes in ion channel function [11], [33]. However, we have shown that injury can be induced by applying small strains, less than what can disrupt the cell membrane, at high rates directly through mechanically sensitive FACs. A recent *in vitro* study directly linked focal swelling to the pathological influx of calcium and activation of calpains which degrade the cytoskeleton [9]. Other studies have shown that not all neuronal injury is dependent on membrane disruption and calpain activity [10], [26], but offer little evidence for an alternative mechanism to account for the calpain-independent injury. Our *in-vitro* study indicates that integrin mediated Rho-ROCK activation may account for calpain independent pathways of injury.

Integrins are expressed heterogeneously throughout the brain and have been shown to be differentially expressed in the adult rat brain [15], [34]. Integrins are highly expressed in synaptic regions [35], [36] and can modulate synaptic plasticity by regulating ion channel currents [16], [20], [37]. In the developing nervous system, integrins are involved in dendrite and axon outgrowth [38], [39], [40], [41] and guide synaptogenesis [20], [37], and in mature neurons, they play a role in remodeling dendritric spines [37], [42]. Their ability to modify Ca^2+^ handling and modulate synaptic strength has also been linked to stabilizing long term memory potentiation [43], suggesting that integrins may be key players in memory and learning [15], [20]. In this study, we showed that axons may be more vulnerable to injury than the soma because the failure strength of FACs in neurites is significantly lower than in the soma. Furthermore, neuronal injury was dependent upon FAC density, and force transmission via integrin binding proteins always produced widespread focal swelling, whereas non-specific force transmission through the membrane produced only local injury. A previous study has demonstrated a similar sensitivity of neuronal injury to ECM composition in the 3D cell microenvironment [43]. Neurons embedded in a 3-D gel composed of collagen conjugated to agarose exhibited increased cell death following an acute, high rate deformation when the collagen concentration was increased, indicating that the degree of cell-ECM contacts may influence neuronal injury [44]. In another study, the threshold for mechanically induced action potentials was found to be lower in neurons cultured on FN compared to those cultured on PLL, underscoring the important role of cell-ECM contacts in neurons [45]. Cell-matrix interactions have also been shown to be involved in pathological processes following acute mechanical stimulation in other cell types such as vascular smooth muscle cells [46] and epithelial cells [47], [48]. These reports, coupled with the data reported herein, suggest integrins are a reasonable conduit for mechanical cell trauma.

Previous reports suggest a role for calpains in neuronal injury [9], [10], [26]. In our low strain model, we were unable to mitigate neuronal injury with a calpain inhibitor. However, we were successful in reducing neurite injury with the use of a ROCK inhibitor. Integrin stimulation can activate many signaling cascades [49], but activation of the Rho-ROCK pathway is of particular interest because of its known effects on the cell cytoskeleton. ROCK activation can affect cytoskeleton remodeling by activating downstream targets which regulate cytoskeleton tension [50], actin polymerization [51], neurofilament depolymerization [52], and microtubule stability [53]. Interestingly, studies have shown that axon focal swelling may be a result of the breakdown of microtubules and impairment of the axonal transport system [54]. Furthermore, axon retraction following mTBI can be linked to active remodeling of the neuronal cytoskeleton [55]. The activation of RhoA in *in vitro* studies has demonstrated neurite retraction in neuroblastoma cell lines [56] and dendritic retraction in brain slices [57]. A genetic study in *Drosophila* indicates that in mature neurons, the RhoA-mediated axon retraction pathway is actively repressed by negative regulators [58]. The synaptic degeneration associated with DAI implies that the activation of RhoA is a maladaptive response. Blocking activation with a Rho antagonist can reduce injury related apoptosis in the CNS [59], suggesting that blocking Rho activation may be effective in treating TBI. Furthermore, recent studies on axon growth cone retraction have demonstrated a link between ECM protein type, integrin activation, cyclic AMP levels, and Rho activity [60]. With the growing concern about the lack of therapeutic options for treating mTBI [61], our results suggest that further exploration of integrin mediated neuronal injury may identify novel therapeutic opportunities.

## Materials and Methods

### Ethics Statement

All procedures were approved by the Harvard Animal Care and Use Committee under Animal Experimentation Protocol permit number 24-01. This protocol, entitled "Harvest and Culture of Neural and Cardiac Tissue from Neonatal Rats and Mice for In Vitro Disease Models," meets the guidelines for the use of vertebrate animals in research and teaching of the Faculty of Arts and Sciences of Harvard University. It also follows recommendations included in the NIH Guide for the care and use of laboratory animals and is in accordance with existing Federal (9 CFR Parts 1,2&3), state and city laws and regulations governing the use of animals in research and teaching.

### Neuron Harvest and Culture

Cortical neurons were isolated from 2-day old neonatal Sprague-Dawley rats (Charles River Laboratories, Boston, MA). Reagents were obtained from Sigma-Aldrich (St. Louis, MO) unless otherwise indicated. Cortices were surgically isolated and minced in Hanks' balanced salt solution (Invitrogen, Carlsbad, CA) followed by digestion with trypsin (USB, Santa Clara, CA) overnight at 4°C. The cell suspension was then filtered though a nylon filter of 40 µm pore size (BD Bioscience) and finally separated using a Percoll gradient (GE Healthcare Life Sciences, Piscataway, NJ). Subsequently, cells were re-suspended in DMEM culture medium (Invitrogen) supplemented with 10% (v/v) heat-inactivated fetal bovine serum (Invitrogen), 30 mM Glucose, 2 mM L-glutamine, 25 mM KCl, 50 mU Insulin, 7 µM *p* -Aminobenzoic acid, 100 U/mL penicillin, and 100 µg/mL streptomycin. Cells were seeded at a density of 30,000 cells per cm^2^ and supplemented with 10 µM cytosine arabinoside for the first 48 hours of culture on substrates coated with either 100 µg/ml PLL or 50 µg/ml FN. Samples were incubated under standard conditions at 37°C and 5% CO_2_. After 48 hours cells were washed 3 times with PBS to remove non-adherent cells. Media was replaced every 48 hours until experiments were executed. All experiments were performed on either day 4 or 5 post seeding.

### High Speed Stretcher *In Vitro* TBI Model

Medical grade silicone elastomer membranes (SMI .010” NRV) were spin-coated with Sylgard 527 (Dow Corning, Midland, MI) polydimethylsiloxane (PDMS) that was mixed at a 1∶1 base to curing agent ratio and allowed to cure for at least 4 hours at 70°C. The elastomer membranes were then clamped into custom made brackets to maintain tension, and a reducing well to hold cell media was adhered using additional PDMS which was allowed to cure again for at least 4 hours at 70°C. Samples were then oxidized using UV ozone (Model No. 342, Jetlight, Irvine, CA) for 8 minutes to sterilize the surface and increase hydrophilicity for protein adsorption. Either isotropic Poly-l-Lysine (PLL) or Fibronectin (FN) (BD Biosciences, San Jose, CA) was then deposited on the PDMS at a concentration of µg/ml or 50 µg/ml, respectively, in sterile deionized water for at least 20 minutes. Excess PLL or FN was removed by washing with deionized water. Neurons were seeded and cultured inside the reducing well as indicated previously. Each sample was loaded into a custom made High Speed Stretching (HSS) device which used a high precision linear motor (LinMot Model P01-23×80F-HP, Elkhorn, WI) to displace the brackets and strain the elastomer sheet to a desired magnitude at a rate of 1% per ms (Movie S1). Membrane strain was verified by recording the deformation of a 1.5×1.5 cm grid using a high speed camera (FasTec Troubleshooter Model #: TS1000ME) and calculating the strain using a three-point strain algorithm [62].

### Immunofluorescent Staining and Microscopy

Cells were washed 3 times in PBS at 37°C and fixed for 10 minutes in 4% paraformaldehyde and 2.5% TritonX-100 in PBS at 37°C. Cells were then washed 3 times in PBS and an initial blocking step using 5% Bovine Serum Albumin (Jackson ImmunoResearch, West Grove, PA) in PBS was performed for 1 hour at 37°C. The blocking solution was aspirated away and the primary antibody solution was immediately added and incubated for 1.5 hours at room temperature. The primary antibodies used were either anti-β-Tubulin III (1∶200), monoclonal anti-Vinculin (1∶200), or anti-glial fibrillary acidic protein (1∶200). Primary antibodies were added to a 0.5% BSA in PBS solution. Following primary staining, cells were washed 3 times, and the secondary staining solution consisting of either goat anti-mouse conjugated to Alexa-Fluor 488 or goat anti-rabbit conjugated to Alexa-Fluor 546 and 4′,6-diamidino-2-phenylindole (DAPI) was added to the cells for 30 minutes at room temperature. Samples were then washed 3 times. For samples seeded on silicon sheets, a scalpel was used to cut out an 18 mm circular section of the substrate which was placed on a glass slide. For glass bottom samples, the glass was removed from the dish and placed on a glass slide. ProLong Gold Antifade reagent (Invitrogen) was added to preserve the samples and glass coverslips are affixed using nail polish (company info). Prepared slides were either imaged immediately or stored at -20°C. Imaging was performed on a LSM 5 LIVE confocal microscope (Carl Zeiss, Oberkochen, Germany) with appropriate filter cubes.

### TUNEL Assay

Click-iT TUNEL assays (Invitrogen) were performed following the manufacturer's protocol [63]. Briefly, cells were fixed in 4% paraformaldehyde and fragmented strands of DNA were labeled with a fluorescent indicator. Fluorescence imaging was performed on experimental and control populations, and neurons exhibiting fluorescence levels above a threshold set by control samples were considered to be apoptotic.

### Membrane Poration Studies

Immediately prior to HSS experiment, membrane impermeable fluorescein dye (Invitrogen) was added to the cell media at a 10 µM concentration. Following completion of the experiment, samples were fixed as described previously but TritonX-100 was excluded. Cell nuclei were labeled with DAPI as described earlier. Uptake of the dye was determined by fluorescence microscopy. Cells exhibiting uptake of the dye above a set fluorescence intensity threshold of three standard deviations greater than the control mean were considered to be permeabilized.

### Magnetic Tweezer Fabrication and Control

The magnetic tweezer was fabricated using a permalloy core (MµShield, Londonderry, NH) that was equipped with a 720-turn solenoid (Magnetic Sensor Systems, Van Nuys, CA). The tweezer ensemble was mounted on an Axio Observer.Z1 microscope (Carl Zeiss) and was controlled by a micromanipulator system (Eppendorf, Hamburg, Germany). Current in the solenoid was produced by a voltage-controlled current supply (Kepco Model # BOP 100-4M, Flushing, NY) that transformed voltage signals from a function generator into a current signal with amplitudes up to 5A. LabVIEW (National Instruments, Austin, TX) software was used to program the desired voltage waveform. The magnetic tweezer was calibrated using methods outlined in [28]. Briefly, beads were placed in a 99% glycerol solution and the tweezer was engaged at various current levels. Utilizing Stoke's formula and magnetic bead velocities, we calculated force as a function of distance from tweezer tip for each current level (fig S4A). Temperature rise of the extracellular media as a result of the Joule effect in the tweezer's coil was determined to be approximately one degree Celsius (fig. S4B). Mechanoporation during bead pull experiments was assessed through rhodamine dye uptake [10] and intracellular rises in calcium concentration (fig. S4C **–**D), revealing no increase in cell membrane permeability.

### Bead Functionalization and Attachment

In order to deliver necessary forces for neuron adhesion strength and peeling experiments, the super paramagnetic beads (Bioclone, San Diego, CA) were coated with fibronectin (10 µg/ml) according to manufacturer's specifications. For bead-induced neuronal injury studies, much less force was required and Dynabeads paramagnetic beads (Invitrogen) were used and coated with either PLL or fibronectin (PLL: 100 µg/ml; FN: 10 µg/ml). For both cases beads were incubated with neurons for a total of 1 hour before experiments.

### Polyacrylamide Gel Fabrication and Functionalization

Cells were cultured on polyacrylamide gels. Acrylamide and bis-acrylamide (Fisher Scientific, Pittsburgh, PA) solutions are prepared to contain a constant polymer mass of 5% and bis-acrylamide concentrations of 0.2% to reach a final modulus of 6 kPa (Brain: E∼0.1**–**1 kPa). Acrylamide, bis-acrylamide, ammonium persulfate, and N,N,N,N-tetramethylethylenediamine (TEMED) under a nonaqueous layer of toluene containing 0.5% acrylic acid N-hydroxy succinimide ester was polymerized between two coverslips, chemically modified as previously described [64]. After washing with HEPES buffer to remove traces of unpolymerized solvent, wells containing the polyacrylamide (PA) gels were filled with sterile water and keep at 4°C until use. The PA gels were coated with poly-L-lysine and fibronectin. Our results clearly show that patterned PA gels create a highly ordered cell matrix for guidance of neurons. We employ micropatterning to control the cell-matrix interaction, to organize neuron cells, and to perform magnetic pulling experiments at the single cell level.

In order to coat the polyacrylamide gel with ECM, a few drops of 1 mM Sulfo-SANPAH (sulfosuccinimidyl-6- (4-azido-2-nitrophenyl-amino) hexanoate); (Pierce/ThermoFisher Scientific, Waltham, MA) in 200 mM HEPES were added to activate the free surface of the gel. The system was then irradiated with the UV light of a sterile hood (254 nm wave length) for 5 min to link the SANPAH to the gel by photoactivation. The solution containing excess SANPAH was removed by aspiration, and the process of adding SANPAH and exposing to vacuum and UV light was repeated once more. In order to maximize the efficiency of FN or PLL transfer to the surface of the PA gel in the desired pattern of 10 µm wide lines, PDMS microfluidic chambers were used to constrain FN or PLL solution on the gel overnight. After the excess SANPAH was again removed by aspiration, the PDMS chamber was gently placed onto the activated polyacrylamide gel. 500 µL of fibronectin or poly-L-lysine solution was added to the edges of the chamber (fig S10). The chambers were constructed so that ECM coating could be sucked in from the sides of the chamber. The ECM protein was held in place by the chamber on the surface of the activated gel overnight at 4°C and excess of solution was removed by washing the gels.

### Focal Adhesion and Vinculin Puncta Quantification

The preparations were immunostained for vinculin, a focal adhesion protein. With fluorescent microscopy, the vinculin plaques representing FACs could be counted and their area, indicating the size of the focal adhesion, calculated within the acquired images. Using a watershed algorithm to separate structures according to the intensity drop between them, individual FACs could be identified and their respective area quantified. In order to segment very large and granular adhesion sites, as observed on FN- and PLL-coated substrates respectively, we used a different set of threshold values for watershed segmentation [65], [66].

### Neuronal Adhesion and Peeling Experiments

In the peeling experiments, the distance between the tip of the tweezer and the bead was maintained at a range of 10**–**30 µm with a micromanipulator, and the current was maintained at 0**–**5 Amps, corresponding to a maximum force magnitude of 30 nN. Cells were imaged in phase contrast with an Axiovert 200 inverted optical microscope (Carl Zeiss) and recorded using a Cascade 512B CCD Camera (Roper Scientific, AZ) in order to determine peeling and detachment. All experiments were conducted in normal Tyrode's solution at 37°C.

### Neuronal Magnetic Tweezer Injury Experiments

Cells were plated onto PDMS-coated coverslips coated with 100 µg/mL Poly-L-Lysine. Cells were loaded with Fluo-4 (Invitrogen) so that calcium activity during the pull could be measured. The magnetic tweezers captured individual beads and applied a short 100 ms pulse between 0.5nN and 5.5nN. Each cell was imaged every minute following bead pull to determine injury outcome. All experiments were conducted in normal Tyrode's solution at 37°C.

### Magnetic Tweezer Membrane Poration Studies

Normal Tyrode's solution was supplemented with 12.5 µM carboxytetramethylrhodamine dye (Invitrogen). Cells were imaged during and after bead pull to assess changes in intracellular dye concentration as an indicator of cell membrane poration, which would allow the dye to rush into the cell. Given the high ratio of extracellular to intracellular calcium concentration, Fluo-4 (Invitrogen) was also used to determine if smaller pores-those that could let calcium ions pass- formed by checking for a rise in intracellular calcium concentration during the bead pull.

### Scanning Electron Microscopy

After beads were attached to neurons they were rinsed twice with PBS fixed with 2.5% Glutaraldehyde for 2 hours. After rinsing again, cells were treated with 1% OsO_4_ for 2 hours. After another rinse with PBS, cells were gradually dehydrated with increasing dilutions of ethanol up to 100%. After critical point drying was completed, cells were gold sputter coated for 2 minutes at 30 mA. All imaging was done on Quanta 200 scanning electron microscopy (FEI, Hillsboro, OR).

### Pharmacological Interventions

Calpain inhibitor MDL 28170 (Sigma) was prepared per manufacturer's recommendations. Briefly, MDL 28170 was reconstituted in anhydrous DMSO and neurons were treated with concentrations ranging between 100 nM and 1 mM in Tyrode's solution. The effects of the calpain inhibitor were determined for both 30 minute pre-incubations and immediate post stretch applications. Rho-associated Kinase (ROCK) inhibitor HA-1077 (Sigma) was prepared by dissolving in water and neurons were treated with concentration ranging between 1 nM and 100 µM in Tyrode's solution. The effects of calpain inhibitor were determined for immediate post stretch applications.

### Statistical Analysis

Statistical significance was measured by ANOVA and subsequent pairwise comparison when comparing multiple values. Fisher's exact test was used to analyze data in contingency tables [67]. p<0.05 for all statistically significant differences.

## Supporting Information

Figure S1 Custom built high speed stretching device delivers precision strain at high rates to an elastomer membrane. (**A**) Image of the device shows culturing well adhered to elastomer membrane which is clamped into mounts and displaced by a linear motor. (**B**) Representative longitudinal 3-point Lagrange strain profiles were measured by high speed imaging of the deformation of a 1.5×1.5 cm grid located at the center of the culturing well. (**C**) Strain fields were found to be uniform in the center of this region (dashed circle).
(TIF)

Figure S2 (**A**) TUNEL staining was performed to detect traumatic DNA fragmentation pursuant to apoptosis at 10 minutes following abrupt 5% and 10% strain, but no significant increase was observed (*n* = 3). (**B**) The amount of apoptotic neurons did not show a significant increase at 60 minutes (*n* = 3; all bars SEM for all panels).
(TIF)

Figure S3 An inverted immunofluorescent image of vinculin puncta from a neuron cultured on a 10 µm wide line of PLL indicates the presence of FACs.
(TIF)

Figure S4 (**A**) Force calibration of 4.5 µm paramagnetic beads was conducted in 99% glycerol solution. The bead velocity was tracked and force was deduced through Stokes formula for low Reynolds flow. (**B**) Induction of magnetic field in tweezers did not result in a large temperature increase after a 1 second 5 Ampere pulse. (**C–D**) Pulling beads bound to neurons did not cause an increase in membrane permeability as evidenced by the lack of rhodamine dye and calcium ion entry into the cell during and after the injury pull.
(TIF)

Figure S5 Time series depicts focal swelling development due to a 3nN 100 ms injury pulse applied at the red paramagnetic bead locations. Focal swellings occurred globally and in bi-directional fashion despite the focal nature of the injury.
(TIF)

Figure S6 (**A**) Panels depict the formation of focal swellings along neurites as seen by phase contrast microscopy following a 1 second 3nN pull on a bound bead. (**B**) Exposing a neuron to the magnetic field alone did not induce an injury. (**C**) The beads alone failed to produce injury without the presence of the magnetic field.
(TIF)

Figure S7 FN-coated paramagnetic beads were attached to neurons as previously described. With no magnetic field, cells did not show signs of injury as indicated by the lack of focal swellings, Ca^2+^ uptake, and Sytox uptake.
(TIF)

Figure S8 Beads coated with Ac-LDL bind neurons nonspecifically through lipid interactions. Since focal adhesions did not form at the bead binding site, force applied to such beads failed to produce injury as indicated by the lack of focal swellings, Ca^2+^ uptake, and Sytox uptake.
(TIF)

Figure S9 Prophylactic treatment with Calpain inhibitor MDL 28170 (30 minutes prior to abrupt strain) applied over a range of concentrations to neurons cultured on PLL was unable to decrease neuronal injury 10 minutes after stretch (*n*≥4; all bars SEM).
(TIF)

Figure S10 In order to pattern 10 µm wide lines on Polyacrylamide (PA) gels, a modified PDMS microfluidic chamber was first placed on top of the gel. Vacuum was then applied to the top of the PDMS chamber through a port connected to the surface features. This vacuum drew ECM solution from the sides of chamber into feature cavities. The PDMS chamber was incubated with the PA gels overnight to ensure ECM transfer.
(TIF)

Video S1 **High Speed Stretcher.** Sample video shows rapid displacement of HSS system used to deliver mechanical stimulation to neuronal cultures.
(AVI)

Video S2 **Global Injury.** Inverted calcium time lapse imaging of neuron after pulled FN-coated bead (red dot indicates bead location). Note retraction of neurites (red arrow) and global extent of injury despite a focal pull. Focal swellings also appear along previously smooth neurites.
(AVI)

Video S3 **Local Injury.** Inverted calcium time lapse imaging of neuron after pulled PLL-coated bead (red dot indicates bead location). Injury is not global but is localized to neurites near the pulled bead (red arrow).
(AVI)

## References

[1] Bhattacharjee Y (2008). Neuroscience. Shell shock revisited: solving the puzzle of blast trauma.. Science.

[2] Hoge CW, McGurk D, Thomas JL, Cox AL, Engel CC (2008). Mild traumatic brain injury in U.S. Soldiers returning from Iraq.. N Engl J Med.

[3] Elder GA, Cristian A (2009). Blast-related mild traumatic brain injury: mechanisms of injury and impact on clinical care.. Mount Sinai Journal of Medicine: A Journal of Translational and Personalized Medicine.

[4] Cernak I, Wang ZG, Jiang JX, Bian XW, Savic J (2001). Ultrastructural and functional characteristics of blast injury-induced neurotrauma.. Journal of Trauma-Injury Infection and Critical Care.

[5] Gennarelli TA, Thibault LE, Adams JH, Graham DI, Thompson CJ (1982). Diffuse axonal injury and traumatic coma in the primate.. Annals of Neurology.

[6] Sponheim SR, McGuire KA, Kang SS, Davenport ND, Aviyente S (2011). Evidence of disrupted functional connectivity in the brain after combat-related blast injury.. NeuroImage.

[7] Mac Donald CL, Johnson AM, Cooper D, Nelson EC, Werner NJ (2011). Detection of blast-related traumatic brain injury in U.S. military personnel.. N Engl J Med.

[8] Mac Donald CL, Dikranian K, Song SK, Bayly PV, Holtzman DM (2007). Detection of traumatic axonal injury with diffusion tensor imaging in a mouse model of traumatic brain injury.. Experimental Neurology.

[9] Kilinc D, Gallo G, Barbee KA (2009). Mechanical membrane injury induces axonal beading through localized activation of calpain.. Exp Neurol.

[10] Farkas O, Lifshitz J, Povlishock JT (2006). Mechanoporation induced by diffuse traumatic brain injury: an irreversible or reversible response to injury?. J Neurosci.

[11] Spaethling JM, Klein DM, Singh P, Meaney DF (2008). Calcium-permeable AMPA receptors appear in cortical neurons after traumatic mechanical injury and contribute to neuronal fate.. J Neurotrauma.

[12] Wang N, Naruse K, Stamenovic D, Fredberg JJ, Mijailovich SM (2001). Mechanical behavior in living cells consistent with the tensegrity model.. Proc Natl Acad Sci U S A.

[13] Jaalouk DE, Lammerding J (2009). Mechanotransduction gone awry.. Nat Rev Mol Cell Biol.

[14] Meyer CJ, Alenghat FJ, Rim P, Fong JH, Fabry B (2000). Mechanical control of cyclic AMP signalling and gene transcription through integrins.. Nat Cell Biol.

[15] Chan CS, Weeber EJ, Kurup S, Sweatt JD, Davis RL (2003). Integrin requirement for hippocampal synaptic plasticity and spatial memory.. J Neurosci.

[16] Lin CY, Hilgenberg LG, Smith MA, Lynch G, Gall CM (2008). Integrin regulation of cytoplasmic calcium in excitatory neurons depends upon glutamate receptors and release from intracellular stores.. Mol Cell Neurosci.

[17] Milner R, Campbell IL (2002). The integrin family of cell adhesion molecules has multiple functions within the CNS.. J Neurosci Res.

[18] Schmid RS, Anton ES (2003). Role of integrins in the development of the cerebral cortex.. Cereb Cortex.

[19] Su L, Lv X, Miao J (2008). Integrin beta 4 in neural cells.. Neuromolecular Med.

[20] Watson PM, Humphries MJ, Relton J, Rothwell NJ, Verkhratsky A (2007). Integrin-binding RGD peptides induce rapid intracellular calcium increases and MAPK signaling in cortical neurons.. Mol Cell Neurosci.

[21] Ellis EF, McKinney JS, Willoughby KA, Liang S, Povlishock JT (1995). A new model for rapid stretch-induced injury of cells in culture: characterization of the model using astrocytes.. J Neurotrauma.

[22] Povlishock J, Christman C (1995). The pathobiology of traumatically induced axonal injury in animals and humans: a review of current thoughts.. Journal of neurotrauma.

[23] Stevens GR, Zhang C, Berg MM, Lambert MP, Barber K (1996). CNS neuronal focal adhesion kinase forms clusters that co-localize with vinculin.. J Neurosci Res.

[24] Fass JN, Odde DJ (2004). Tensile force-dependent neurite elicitation via anti-beta 1 integrin antibody-coated magnetic beads. (vol 85, pg 623, 2003).. Biophysical Journal.

[25] Buki A, Farkas O, Doczi T, Povlishock JT (2003). Preinjury administration of the calpain inhibitor MDL-28170 attenuates traumatically induced axonal injury.. Journal of neurotrauma.

[26] Stone JR, Okonkwo DO, Dialo AO, Rubin DG, Mutlu LK (2004). Impaired axonal transport and altered axolemmal permeability occur in distinct populations of damaged axons following traumatic brain injury.. Experimental Neurology.

[27] Marmarou CR, Walker SA, Davis CL, Povlishock JT (2005). Quantitative analysis of the relationship between intra-axonal neurofilament compaction and impaired axonal transport following diffuse traumatic brain injury.. Journal of neurotrauma.

[28] Matthews BD, Overby DR, Mannix R, Ingber DE (2006). Cellular adaptation to mechanical stress: role of integrins, Rho, cytoskeletal tension and mechanosensitive ion channels.. J Cell Sci.

[29] Mitra S, Hanson D, Schlaepfer D (2005). Focal adhesion kinase: in command and control of cell motility.. Nature Reviews Molecular Cell Biology.

[30] Dubreuil CI, Marklund N, Deschamps K, McIntosh TK, McKerracher L (2006). Activation of Rho after traumatic brain injury and seizure in rats.. Exp Neurol.

[31] Mueller B, Mack H, Teusch N (2005). Rho kinase, a promising drug target for neurological disorders.. Nature Reviews Drug Discovery.

[32] Geddes DM, Cargill RS, 2nd, LaPlaca MC (2003). Mechanical stretch to neurons results in a strain rate and magnitude-dependent increase in plasma membrane permeability.. J Neurotrauma.

[33] Wolf JA, Stys PK, Lusardi T, Meaney D, Smith DH (2001). Traumatic axonal injury induces calcium influx modulated by tetrodotoxin-sensitive sodium channels.. J Neurosci.

[34] Pinkstaff JK, Detterich J, Lynch G, Gall C (1999). Integrin subunit gene expression is regionally differentiated in adult brain.. J Neurosci.

[35] Rodriguez MA, Pesold C, Liu WS, Kriho V, Guidotti A (2000). Colocalization of integrin receptors and reelin in dendritic spine postsynaptic densities of adult nonhuman primate cortex.. Proc Natl Acad Sci U S A.

[36] Nishimura SL, Boylen KP, Einheber S, Milner TA, Ramos DM (1998). Synaptic and glial localization of the integrin alphavbeta8 in mouse and rat brain.. Brain Res.

[37] Shi Y, Ethell IM (2006). Integrins control dendritic spine plasticity in hippocampal neurons through NMDA receptor and Ca2+/calmodulin-dependent protein kinase II-mediated actin reorganization.. Journal of Neuroscience.

[38] Robles E, Gomez TM (2006). Focal adhesion kinase signaling at sites of integrin-mediated adhesion controls axon pathfinding.. Nat Neurosci.

[39] Tomaselli KJ, Neugebauer KM, Bixby JL, Lilien J, Reichardt LF (1988). N-cadherin and integrins: two receptor systems that mediate neuronal process outgrowth on astrocyte surfaces.. Neuron.

[40] Hoang B, Chiba A (1998). Genetic analysis on the role of integrin during axon guidance in Drosophila.. J Neurosci.

[41] Schmidt CE, Dai J, Lauffenburger DA, Sheetz MP, Horwitz AF (1995). Integrin-cytoskeletal interactions in neuronal growth cones.. J Neurosci.

[42] Webb DJ, Zhang H, Majumdar D, Horwitz AF (2007). alpha5 integrin signaling regulates the formation of spines and synapses in hippocampal neurons.. J Biol Chem.

[43] Staubli U, Chun D, Lynch G (1998). Time-dependent reversal of long-term potentiation by an integrin antagonist.. J Neurosci.

[44] Cullen DK, Lessing MC, LaPlaca MC (2007). Collagen-dependent neurite outgrowth and response to dynamic deformation in three-dimensional neuronal cultures.. Annals of Biomedical Engineering.

[45] Lin YW, Cheng CM, Leduc PR, Chen CC (2009). Understanding sensory nerve mechanotransduction through localized elastomeric matrix control.. PLoS One.

[46] Wernig F, Mayr M, Xu QB (2003). Mechanical stretch-induced apoptosis in smooth muscle cells is mediated by beta(1)-integrin signaling pathways.. Hypertension.

[47] Tzima E, del Pozo MA, Shattil SJ, Chien S, Schwartz MA (2001). Activation of integrins in endothelial cells by fluid shear stress mediates Rho-dependent cytoskeletal alignment.. EMBO J.

[48] Birukova AA, Fu P, Xing J, Yakubov B, Cokic I (2010). Mechanotransduction by GEF-H1 as a novel mechanism of ventilator-induced vascular endothelial permeability.. Am J Physiol Lung Cell Mol Physiol.

[49] Schwartz MA, Shattil SJ (2000). Signaling networks linking integrins and Rho family GTPases.. Trends in Biochemical Sciences.

[50] Kimura K, Ito M, Amano M, Chihara K, Fukata Y (1996). Regulation of Myosin Phosphatase by Rho and Rho-Associated Kinase (Rho- Kinase).. Science.

[51] Ohashi K, Nagata K, Maekawa M, Ishizaki T, Narumiya S (2000). Rho-associated Kinase ROCK Activates LIM-kinase 1 by Phosphorylation at Threonine 508 within the Activation Loop.. Journal of Biological Chemistry.

[52] Hashimoto R, Nakamura Y, Goto H, Wada Y, Sakoda S (1998). Domain- and Site-Specific Phosphorylation of Bovine NF-L by Rho-Associated Kinase.. Biochemical and Biophysical Research Communications.

[53] Amano M, Kaneko T, Maeda A, Nakayama M, Ito M (2003). Identification of Tau and MAP2 as novel substrates of Rho-kinase and myosin phosphatase.. Journal of Neurochemistry.

[54] Kilinc D, Gallo G, Barbee KA (2008). Mechanically-induced membrane poration causes axonal beading and localized cytoskeletal damage.. Experimental Neurology.

[55] Luo L, O'Leary DD (2005). Axon retraction and degeneration in development and disease.. Annu Rev Neurosci.

[56] Jalink K, van Corven EJ, Hengeveld T, Morii N, Narumiya S (1994). Inhibition of lysophosphatidate- and thrombin-induced neurite retraction and neuronal cell rounding by ADP ribosylation of the small GTP-binding protein Rho.. J Cell Biol.

[57] Bito H, Furuyashiki T, Ishihara H, Shibasaki Y, Ohashi K (2000). A critical role for a Rho-associated kinase, p160ROCK, in determining axon outgrowth in mammalian CNS neurons.. Neuron.

[58] Billuart P, Winter CG, Maresh A, Zhao X, Luo L (2001). Regulating axon branch stability: the role of p190 RhoGAP in repressing a retraction signaling pathway.. Cell.

[59] Dubreuil CI, Winton MJ, McKerracher L (2003). Rho activation patterns after spinal cord injury and the role of activated Rho in apoptosis in the central nervous system.. J Cell Biol.

[60] Lemons ML, Condic ML (2006). Combined integrin activation and intracellular cAMP cause Rho GTPase dependent growth cone collapse on laminin-1.. Exp Neurol.

[61] Janowitz T, Menon DK (2010). Exploring new routes for neuroprotective drug development in traumatic brain injury.. Sci Transl Med.

[62] Alford PW, Taber LA (2003). Regional epicardial strain in the embryonic chick heart during the early looping stages.. Journal of Biomechanics.

[63] Nakayama K, Ohkawara T, Hiratochi M, Koh CS, Nagase H (2008). The intracellular domain of amyloid precursor protein induces neuron-specific apoptosis.. Neuroscience letters.

[64] Pelham R, Wang Y (1997). Cell locomotion and focal adhesions are regulated by substrate flexibility.. Proceedings of the National Academy of Sciences of the United States of America.

[65] Kam Z, Zamir E, Geiger B (2001). Probing molecular processes in live cells by quantitative multidimensional microscopy.. TRENDS in Cell Biology.

[66] Zamir E, Katz B, Aota S, Yamada K, Geiger B (1999). Molecular diversity of cell-matrix adhesions.. Journal of Cell Science.

[67] Upton GJG (1992). Fisher's exact test.. Journal of the Royal Statistical Society Series A (Statistics in society).

