# Supplementary figures and images for: A Possible Role for Integrin Signaling in Diffuse Axonal Injury
Source: PLoS One. 2011 Jul 22;6(7):e22899. doi: 10.1371/journal.pone.0022899 (PMC3142195; doi:10.1371/journal.pone.0022899)

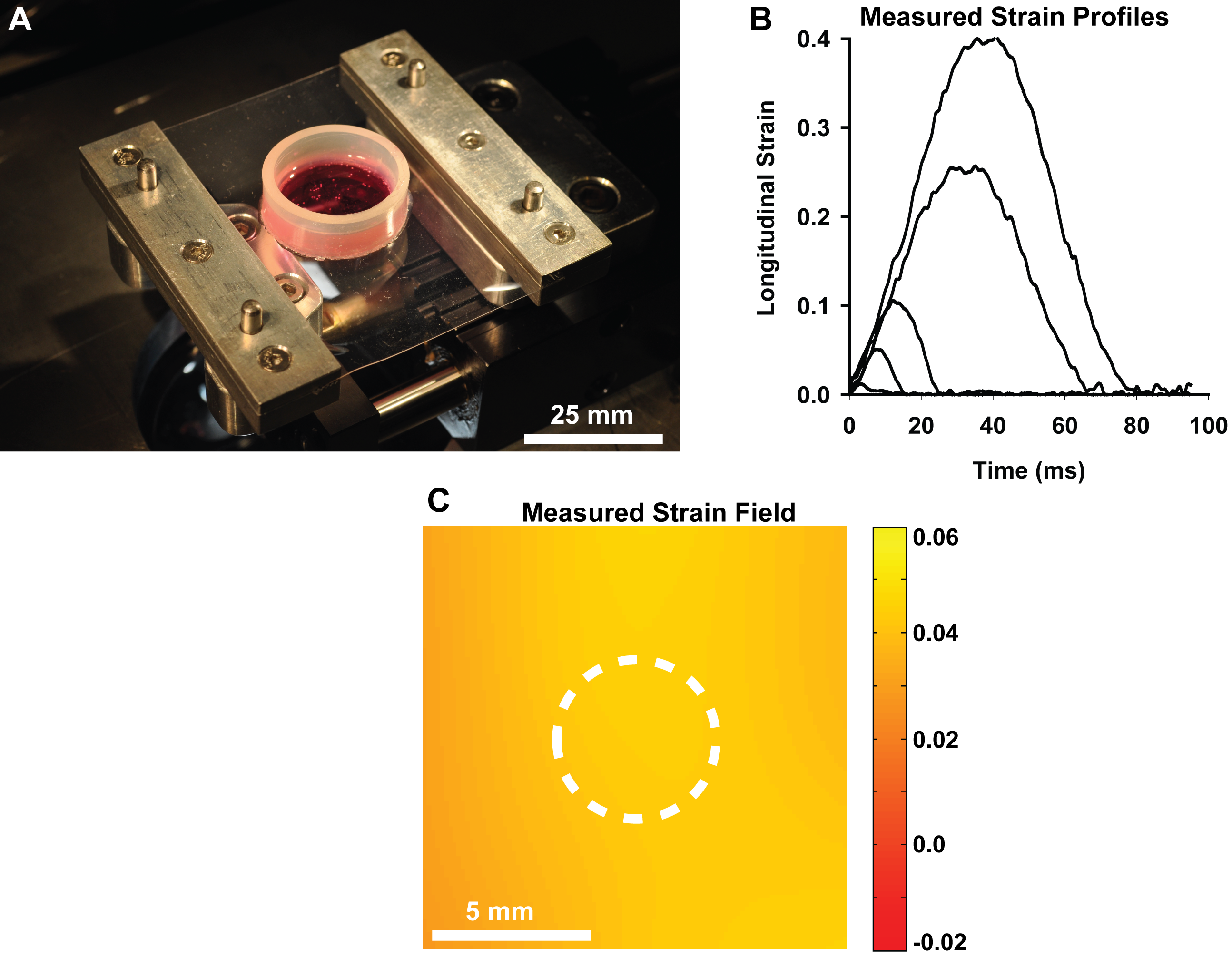

Supplement: Figure S1 — Custom built high speed stretching device delivers precision strain at high rates to an elastomer membrane. (A) Image of the device shows culturing well adhered to elastomer membrane which is clamped into mounts and displaced by a linear motor. (B) Representative longitudinal 3-point Lagrange strain profiles were measured by high speed imaging of the deformation of a 1.5×1.5 cm grid located at the center of the culturing well. (C) Strain fields were found to be uniform in the center of this region (dashed circle). (TIF) [file pone.0022899.s001.tif]

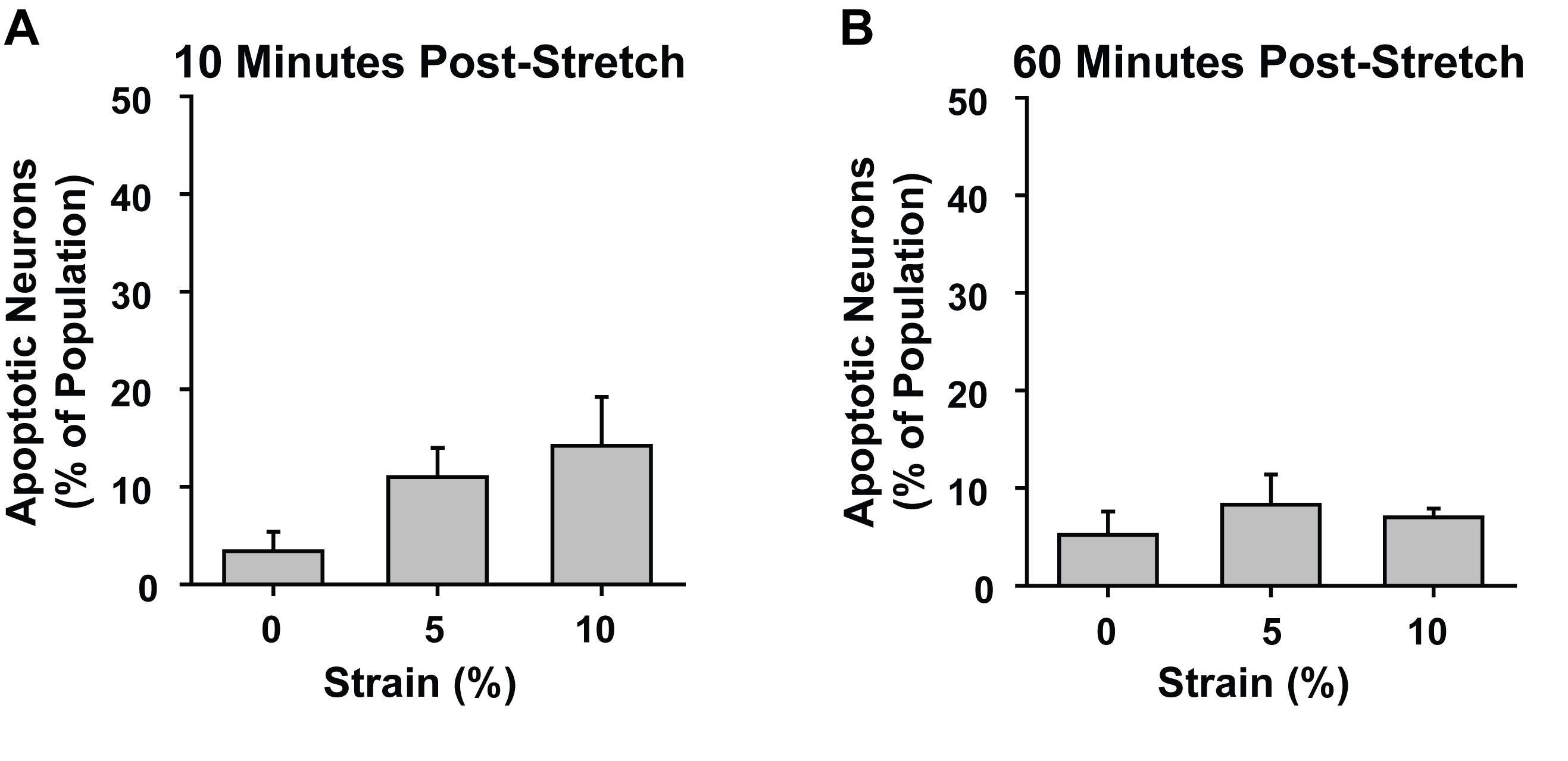

Supplement: Figure S2 — (A) TUNEL staining was performed to detect traumatic DNA fragmentation pursuant to apoptosis at 10 minutes following abrupt 5% and 10% strain, but no significant increase was observed (n = 3). (B) The amount of apoptotic neurons did not show a significant increase at 60 minutes (n = 3; all bars SEM for all panels). (TIF) [file pone.0022899.s002.tif]

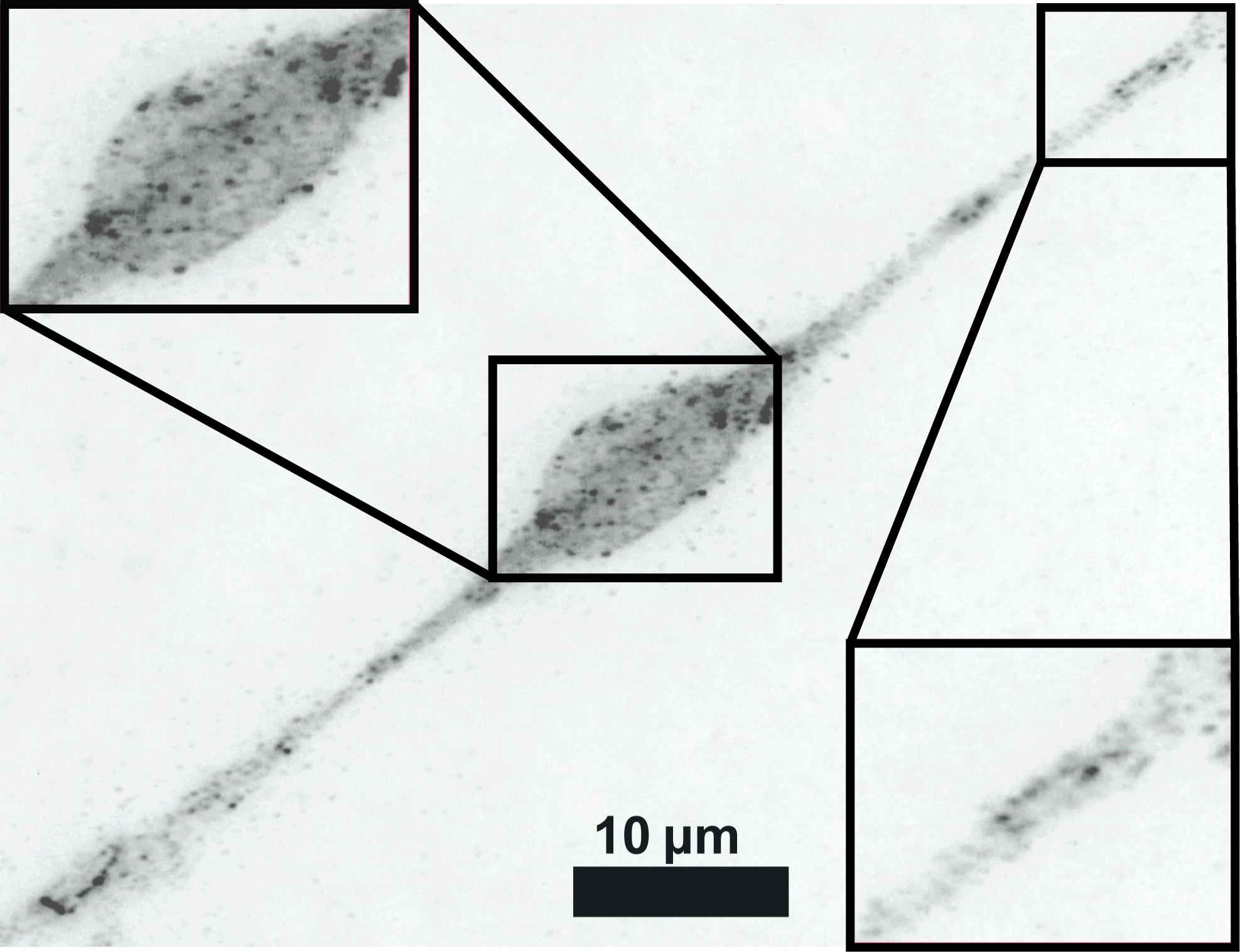

Supplement: Figure S3 — An inverted immunofluorescent image of vinculin puncta from a neuron cultured on a 10 µm wide line of PLL indicates the presence of FACs. (TIF) [file pone.0022899.s003.tif]

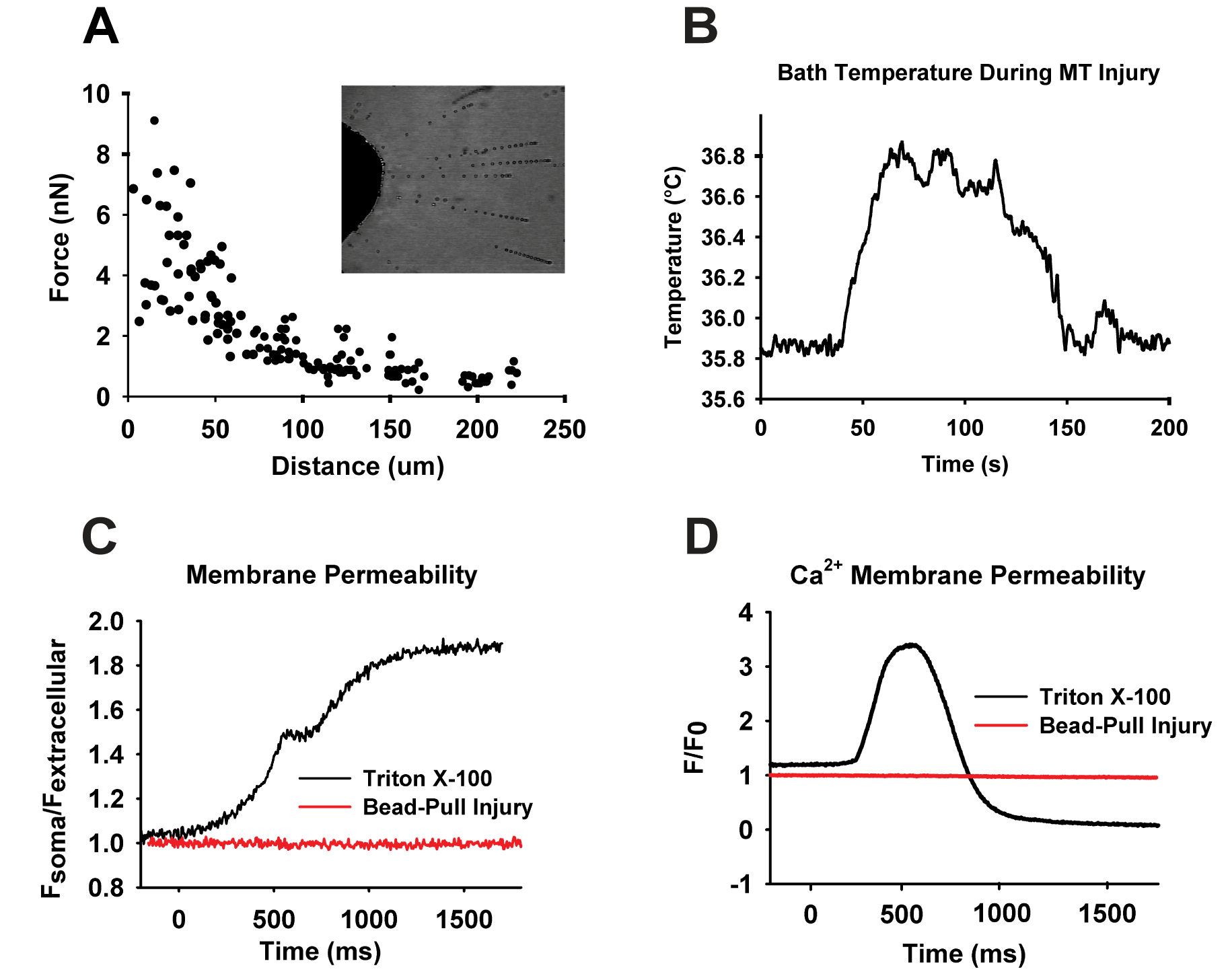

Supplement: Figure S4 — (A) Force calibration of 4.5 µm paramagnetic beads was conducted in 99% glycerol solution. The bead velocity was tracked and force was deduced through Stokes formula for low Reynolds flow. (B) Induction of magnetic field in tweezers did not result in a large temperature increase after a 1 second 5 Ampere pulse. (C–D) Pulling beads bound to neurons did not cause an increase in membrane permeability as evidenced by the lack of rhodamine dye and calcium ion entry into the cell during and after the injury pull. (TIF) [file pone.0022899.s004.tif]

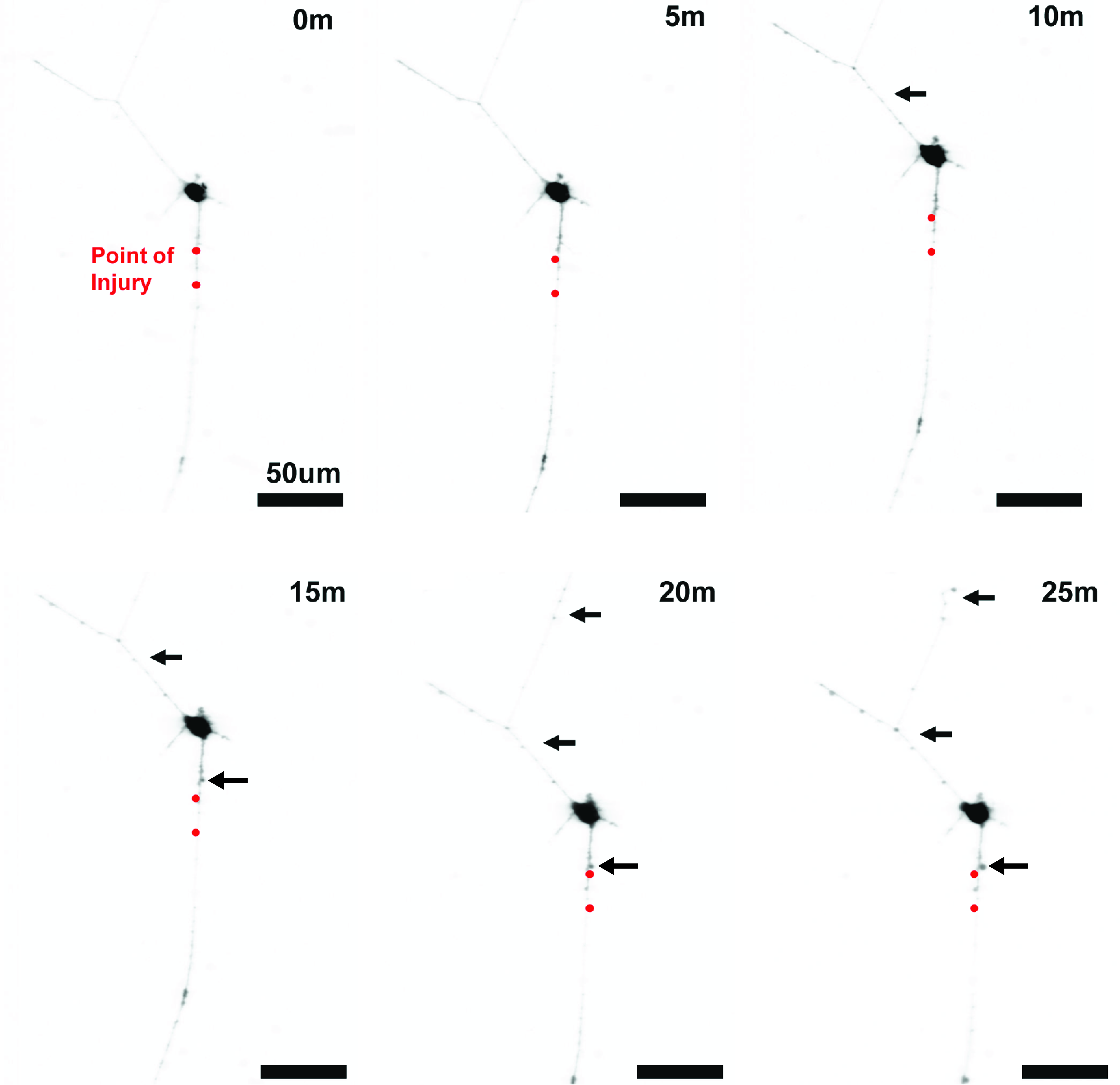

Supplement: Figure S5 — Time series depicts focal swelling development due to a 3nN 100 ms injury pulse applied at the red paramagnetic bead locations. Focal swellings occurred globally and in bi-directional fashion despite the focal nature of the injury. (TIF) [file pone.0022899.s005.tif]

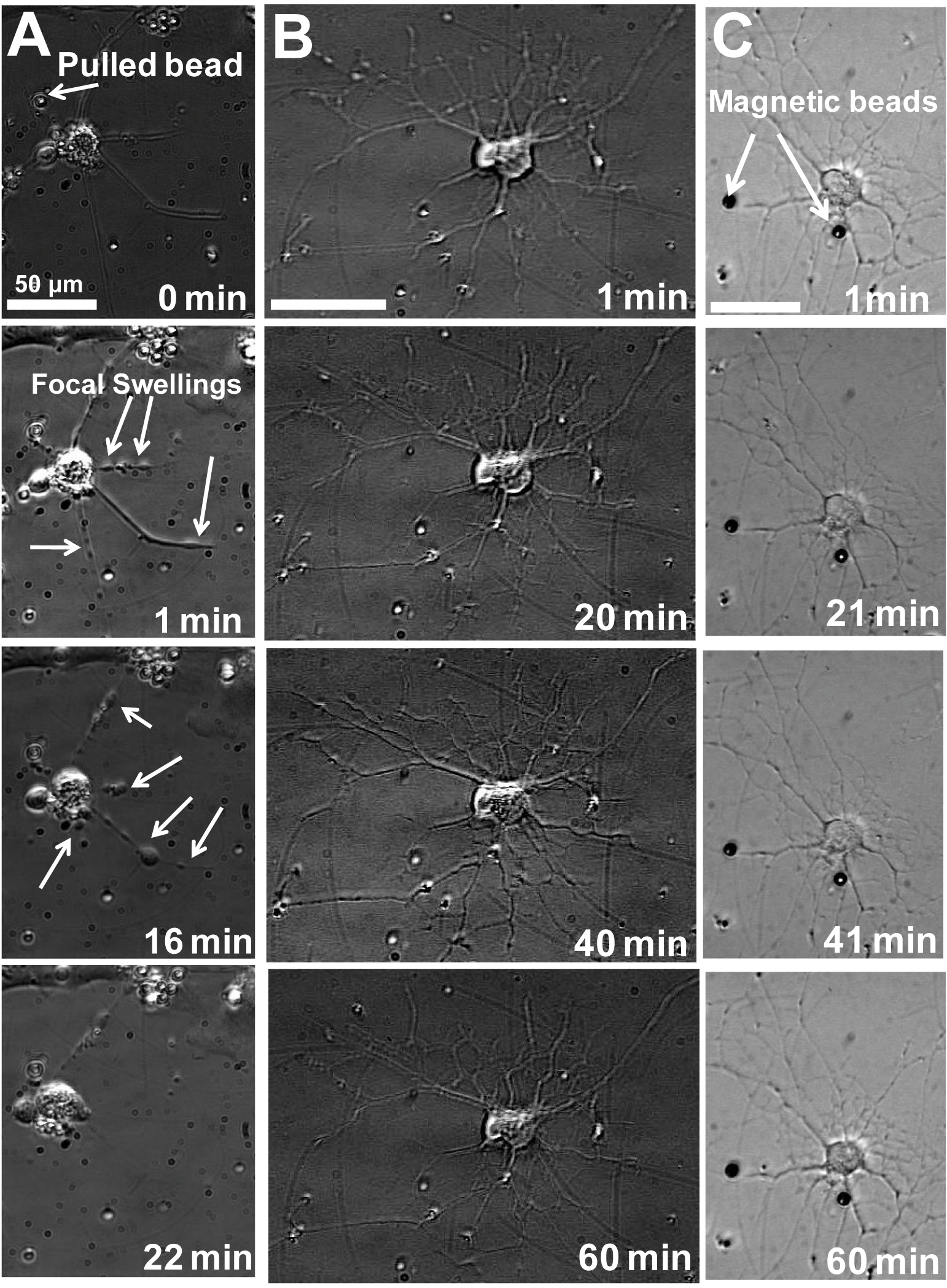

Supplement: Figure S6 — (A) Panels depict the formation of focal swellings along neurites as seen by phase contrast microscopy following a 1 second 3nN pull on a bound bead. (B) Exposing a neuron to the magnetic field alone did not induce an injury. (C) The beads alone failed to produce injury without the presence of the magnetic field. (TIF) [file pone.0022899.s006.tif]

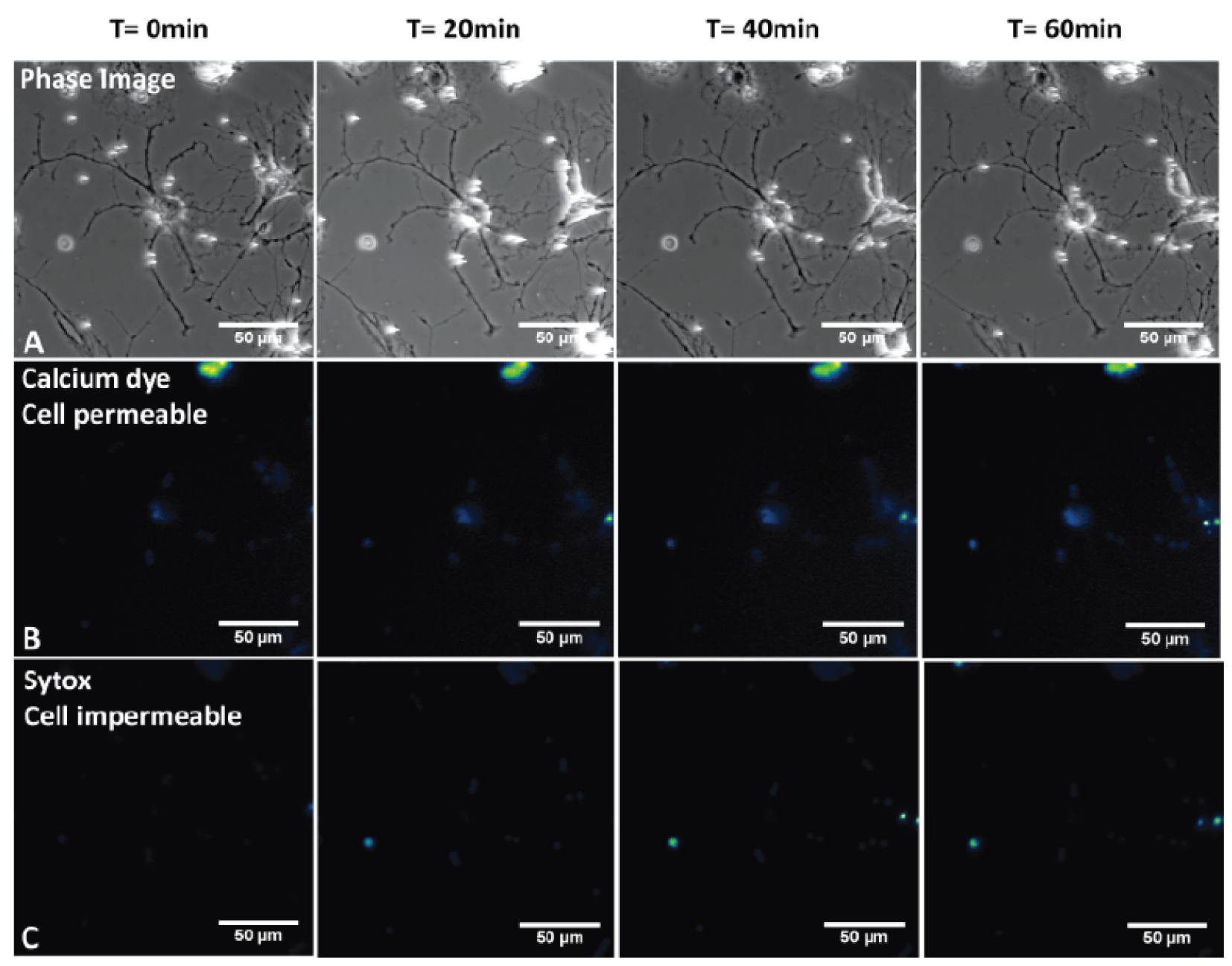

Supplement: Figure S7 — FN-coated paramagnetic beads were attached to neurons as previously described. With no magnetic field, cells did not show signs of injury as indicated by the lack of focal swellings, Ca2+ uptake, and Sytox uptake. (TIF) [file pone.0022899.s007.tif]

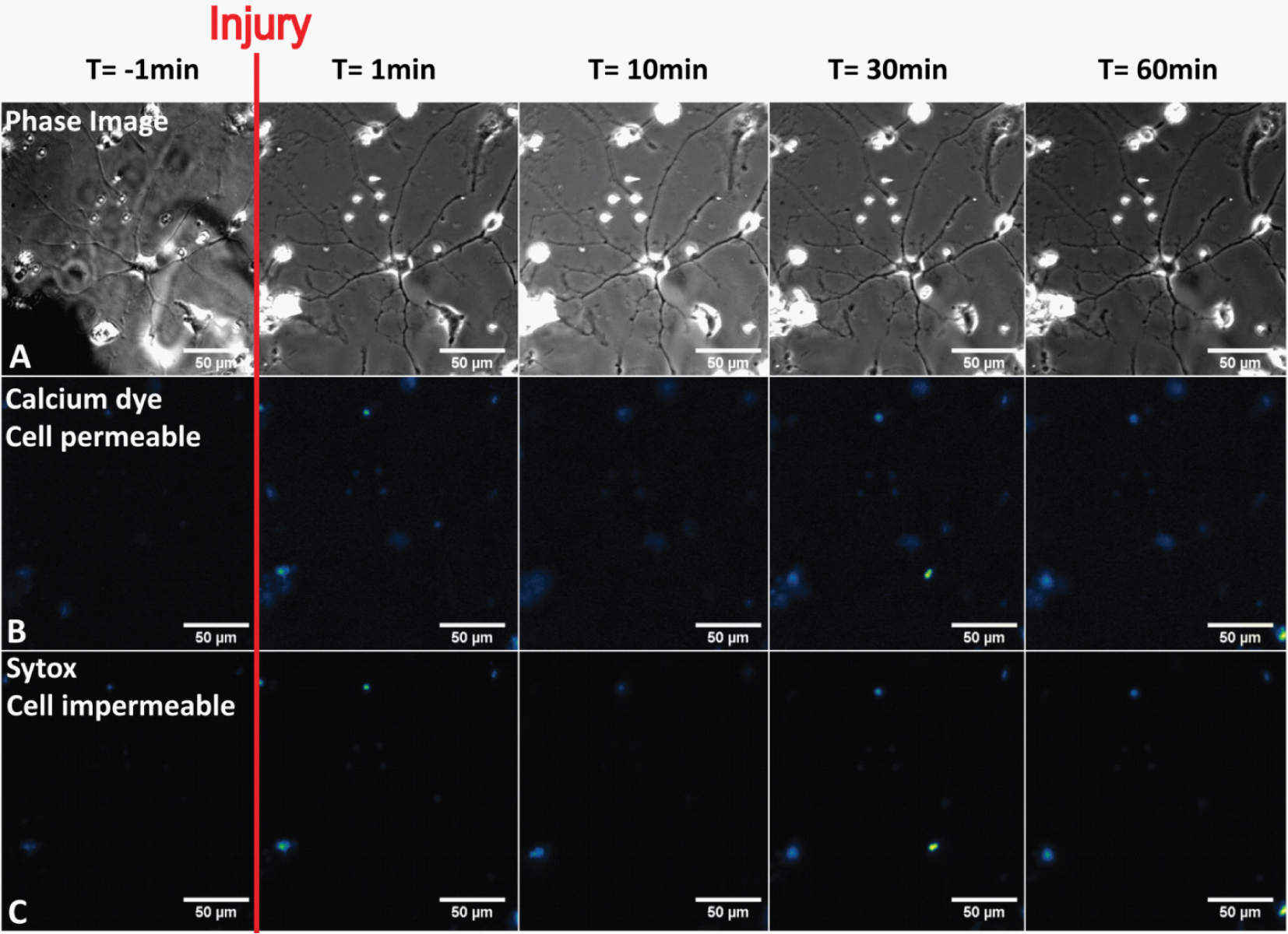

Supplement: Figure S8 — Beads coated with Ac-LDL bind neurons nonspecifically through lipid interactions. Since focal adhesions did not form at the bead binding site, force applied to such beads failed to produce injury as indicated by the lack of focal swellings, Ca2+ uptake, and Sytox uptake. (TIF) [file pone.0022899.s008.tif]

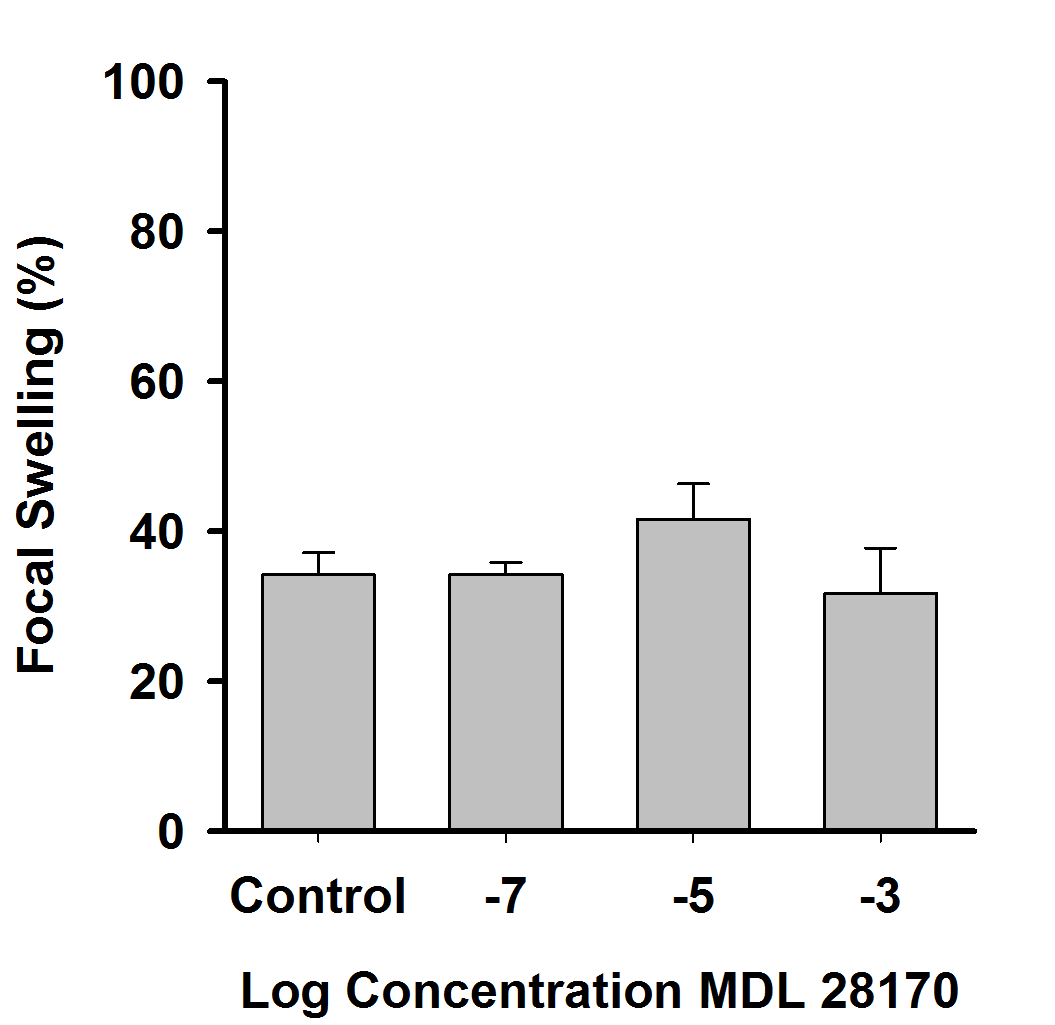

Supplement: Figure S9 — Prophylactic treatment with Calpain inhibitor MDL 28170 (30 minutes prior to abrupt strain) applied over a range of concentrations to neurons cultured on PLL was unable to decrease neuronal injury 10 minutes after stretch (n≥4; all bars SEM). (TIF) [file pone.0022899.s009.tif]

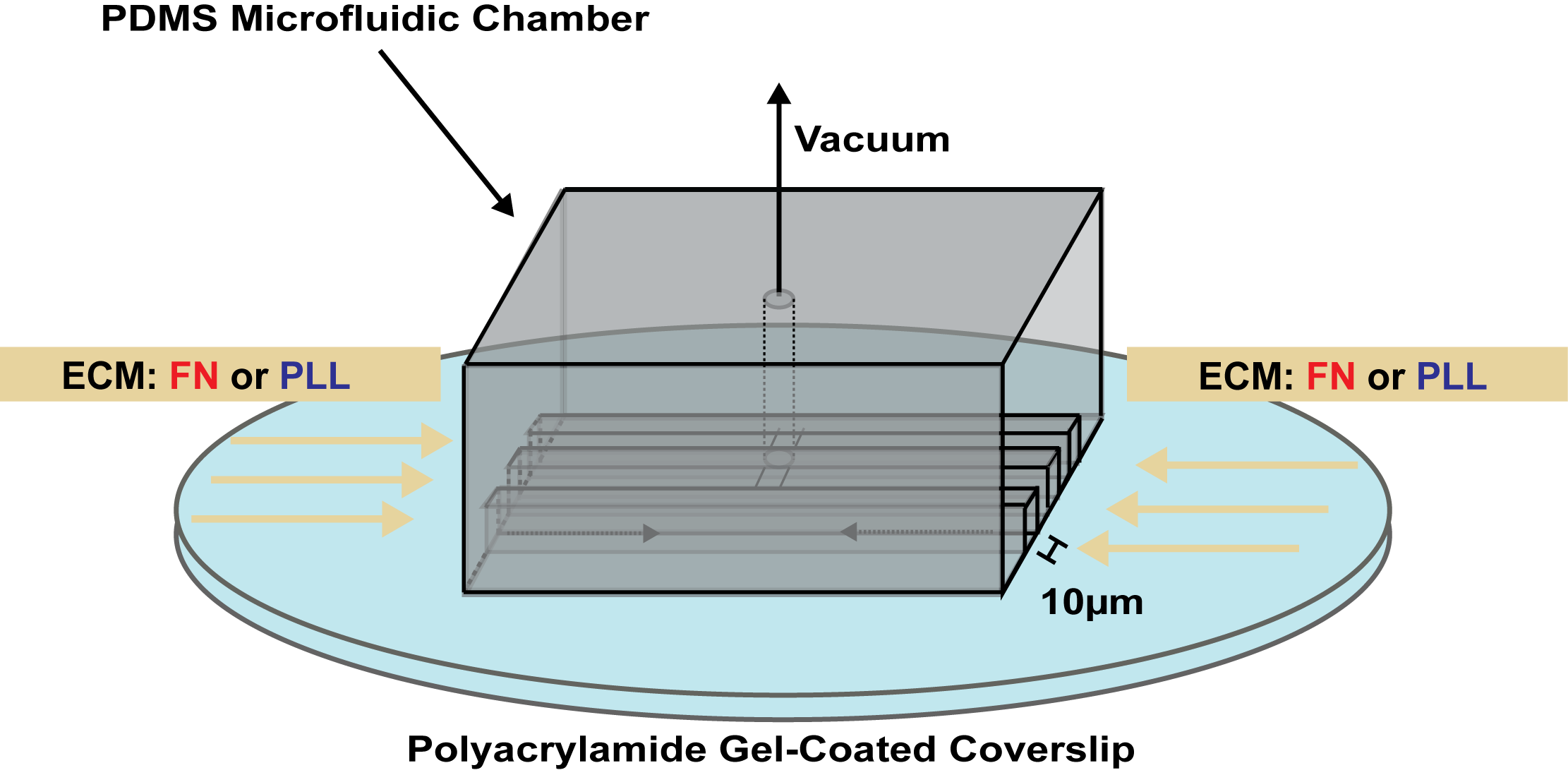

Supplement: Figure S10 — In order to pattern 10 µm wide lines on Polyacrylamide (PA) gels, a modified PDMS microfluidic chamber was first placed on top of the gel. Vacuum was then applied to the top of the PDMS chamber through a port connected to the surface features. This vacuum drew ECM solution from the sides of chamber into feature cavities. The PDMS chamber was incubated with the PA gels overnight to ensure ECM transfer. (TIF) [file pone.0022899.s010.tif]
